# Association of Gestational Weight Trajectories With Neonatal Outcomes Among Pregnant Slum‐Dwelling Women, India

**DOI:** 10.1111/mcn.13805

**Published:** 2025-04-28

**Authors:** Swapna Deshpande, Tarja I. Kinnunen, Rubina Mandlik, Anuradha Khadilkar, Suhas Otiv, Sangita Kulathinal

**Affiliations:** ^1^ Unit of Health Sciences, Faculty of Social Sciences Tampere University Tampere Finland; ^2^ Growth and Paediatric Endocrine Department Hirabai Cowasji Jehangir Medical Research Institute Pune India; ^3^ Interdisciplinary School of Health Sciences Savitribai Phule Pune University Pune India; ^4^ Obstetrics & Gynaecology Department KEM Hospital Pune India; ^5^ Department of Mathematics and Statistics University of Helsinki Helsinki Finland

**Keywords:** India, linear regression K‐means technique, longitudinal clustering, neonatal outcomes, slum‐dwelling pregnant women, weight trajectory

## Abstract

The influence of early pregnancy weight and gestational weight gain (GWG) on neonatal outcomes among Indian slum‐dwellers remains understudied. A prospective cohort study summarised maternal weight trajectories using the longitudinal clustering technique and explored associations between these clusters and neonatal outcomes (low birthweight, small for gestational age [SGA] and preterm births) among 423 pregnant slum‐dwelling women in Pune, India. Sociodemographic data, height and weight were measured at enrolment (< 12 weeks, ‘early pregnancy’). Weight was additionally measured at 23 ± 1 (‘mid‐pregnancy’), 33 ± 1 (‘late pregnancy’), 36–37 and 39–40 weeks. The mean age was 24.7 (95% CI, 23.3, 25.1) years and the mean BMI at enrolment was 22.3 (95% CI, 21.9, 22.7) kg/m^2^. Underweight women had the highest GWG rates and total GWG, while obese women had the lowest. Four clusters were identified: Cluster 1 (*n* = 124, 97% normal and overweight women, GWG rate: 0.27 (95% CI, 0.24, 0.30) kg/week early‐late pregnancy) was the reference group. Women in Cluster 2 (*n* = 146, 93% underweight and normal weight women, GWG rate: 0.31 (95% CI, 0.28, 0.34) kg/week early‐late pregnancy) had a higher risk of having SGA and preterm newborns and women in Cluster 3 (*n* = 68, 100% overweight and obese women, GWG rate: 0.17, 95% CI, 0.12, 0.22 kg/week early‐late pregnancy) had a higher risk of having preterm newborns than Cluster 1. The women in Cluster 4 (*n* = 85, 100% underweight and normal weight, mean early‐late pregnancy GWG rate of 0.47, 95% CI, 0.44, 0.50 kg/week) showed no higher risk of adverse neonatal outcomes. This study highlights the need to monitor both pre‐pregnancy BMI and weight throughout pregnancy to enhance the possibility of favourable neonatal outcomes.

## Summary

- The mean rates of gestational weight gain at mid‐ and late‐pregnancy, as well as total GWG, were highest among underweight women and lowest among obese women in this cohort of urban slum‐dwelling women.
- Our study highlights the need to address both pre‐pregnancy BMI and regular weight monitoring throughout pregnancy to enhance the likelihood of favourable neonatal outcomes.
- Given the pre‐pregnancy weight and weight during the first trimester, we can predict the weight trajectory for the later part of the pregnancy and predict the risk of adverse neonatal outcomes. Such predictive modelling can be valuable for counselling expectant mothers. However, to ensure the generalisability of this approach, larger‐scale studies are necessary.

## Graphical abstract

This prospective study among pregnant slum‐dwelling Indian women highlights the need to address both pre‐pregnancy BMI and regular weight monitoring throughout pregnancy to enhance the likelihood of favourable neonatal outcomes.

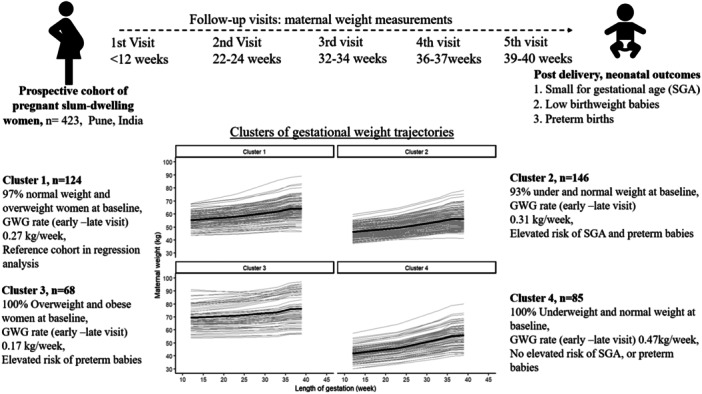

## 1 Introduction

Optimal maternal nutritional status is key to healthy maternofetal outcomes (Victora et al. 2021). Maternal pre‐pregnancy body mass index (BMI) and weight gain during pregnancy influence pregnancy outcomes significantly (Bhavadharini et al. 2017; Cornish et al. 2024; Goldstein et al. 2017). The Institute of Medicine (IOM) guidelines developed for the total gestational weight gain (GWG) and the rate of weight gain by women's pre‐pregnancy BMI were constructed using evidence from the United States (Committee on Obstetric Practice 2013). Insufficient GWG is associated with preterm births, low birth weight (LBW) and small for gestational age (SGA) babies (Goldstein et al. 2017). Excessive GWG is associated with macrosomia, large for gestational age (LGA) babies, caesarean deliveries and post‐partum weight retention (Goldstein et al. 2017).

India, the largest country in South Asia, is grappling with a double burden of malnutrition. In India, the prevalence of underweight (BMI < 18.5 kg/m^2^) is 19% and the prevalence of overweight/obesity (BMI ≥ 25 kg/m^2^) is 24% among women of reproductive age (International Institute for Population Sciences IIPS and ICF [2021]). India contributes the highest number of LBW babies in the world, via the underlying pathways of either born as SGA or born too soon (preterm) babies (Ashorn et al. 2023). A recent national survey estimated that 12% of births in India are preterm, and 18% are LBWs (Jana 2023). LBW babies are at higher risk of neonatal mortality and experience challenges with cognitive capacity and growth faltering in childhood (Christian et al. 2013). Furthermore, the prevalence of macrosomia is reported to be 0.5%, while the prevalence of LGA has ranged between 6.6% and 12% in India (Harvey et al. 2021; Koyanagi et al. 2013). Macrosomia/LGA increases the immediate and long‐term risk for adverse outcomes for the mother and the infant (Rossi et al. 2013; Schellong et al. 2012).

Pregnant Indian women are advised to visit antenatal care (ANC) clinics at least four times during pregnancy, where their weight is measured as a part of their health assessment (World Health Organisation n.d.a). In practice, the timing and number of the ANC visits vary. Assessing the appropriateness of GWG in relation to the risk of neonatal outcomes is challenging in the absence of national GWG guidelines. In addition, weight gain patterns vary among individuals in different periods of pregnancy. There might be subgroups within a population having different patterns of weight gain (Nagin et al. 2018), leading to a comparable total GWG but through different GWG patterns. Modelling individual weight trajectories can reveal unique patterns, identify groups at risk for adverse neonatal outcomes and facilitate targeted interventions (Nagin et al. 2018; Song 2019). Therefore, a data‐driven approach may be used to explore the combined effect of maternal pre‐pregnancy weight and GWG on neonatal outcomes.

Steady urbanisation in India has led to a significant increase in people living in slums (‘a contiguous settlement where the inhabitants are characterised as having inadequate housing and basic services’) (United Nations Human Settlements Programme n.d.). The double burden of malnutrition is an emerging problem in slums (Deshpande et al. 2022; Mamidi et al. 2022; Nguyen et al. 2021); however, the health of slum‐dwelling women is less studied (Ezeh et al. 2017). Few Indian studies (Mamidi et al. 2022; Potdar et al. 2014) have examined the impact of early pregnancy weight and GWG on pregnancy outcomes in this population.

To bridge this gap, we explored the combined effect of early pregnancy maternal BMI status and rate of GWG on neonatal outcomes among slum‐dwellers who are the most vulnerable in the urban context (Ezeh et al. 2017). We used a sound statistical method to summarise the gestational weight measurements collected at different gestational ages. The specific aims of our study were (1) to assess the rates of GWG in mid‐ and late pregnancy and the total GWG according to maternal BMI categories in early pregnancy, (2) to summarise the trajectories of maternal weight using longitudinal clustering techniques and (3) to assess possible association of clusters with neonatal outcomes among pregnant slum‐dwelling women in Pune, India.

## 2 Methods

### 2.1 Study Design and Study Population

We conducted an observational, prospective cohort study (Mother and Infant study) among pregnant women living in slums in Pune, Western India. Using convenience sampling, we chose a public ANC clinic located near a large slum area. Pregnant women were registered at the ANC clinic and visited the facility for routine checkups and delivery. The research field staff explained the study to the women attending these clinics. Women who were willing to participate in the study were screened for eligibility. The eligibility criteria included (a) willingness to participate in the study and ability to give informed consent, (b) residence in a registered slum, (c) gestational age less than 12 weeks, (d) singleton low‐risk pregnancy, (e) absence of major health issues (such as hypertension, diabetes mellitus, tuberculosis or kidney disease) that require intensive hospital follow‐up and (f) intention to attend the same ANC clinic and to give birth in the same health facility or the same city.

**Figure 1:**
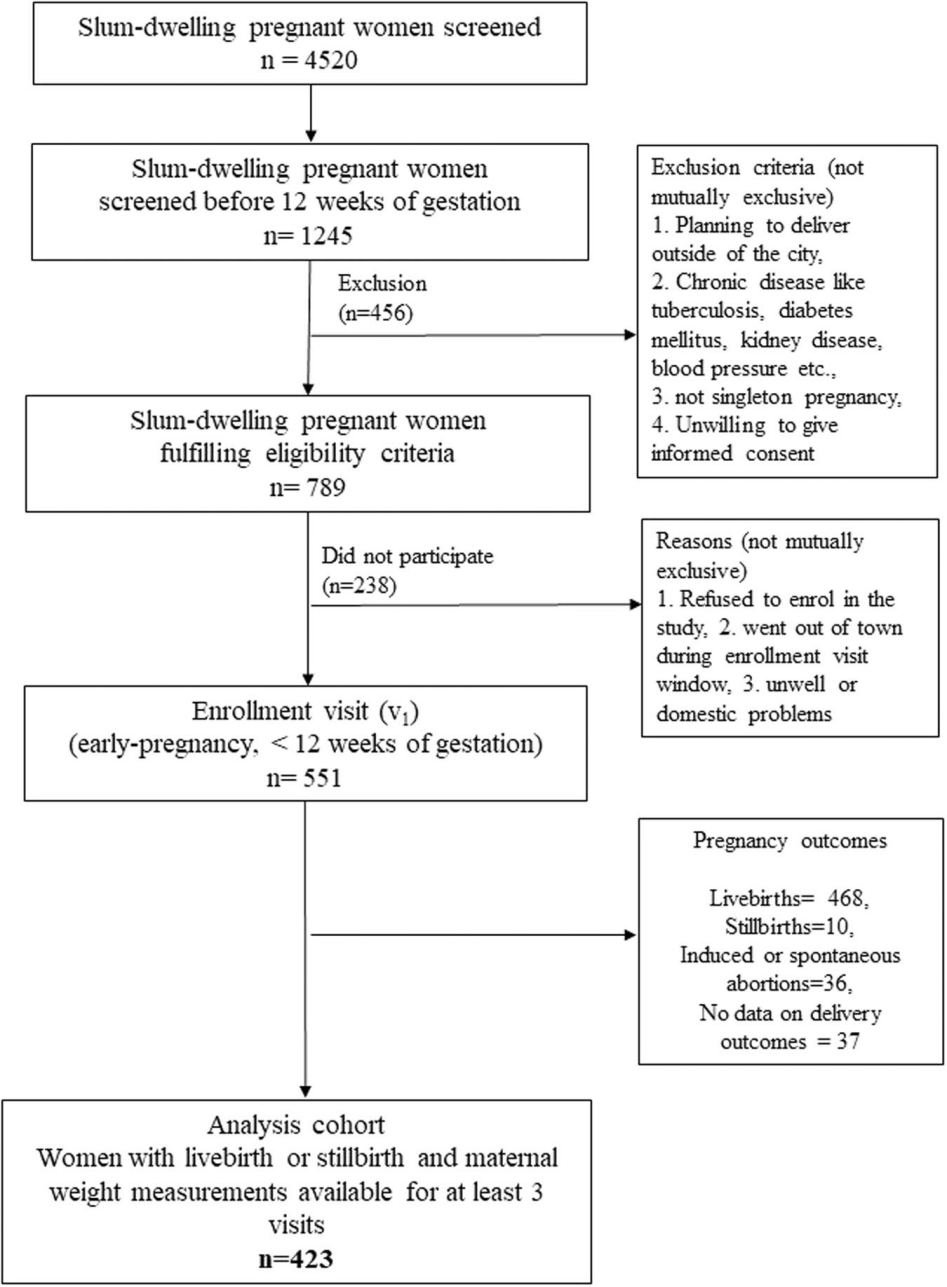
Flowchart of the screening, enrolment and cohort analysis set.

Of the 4520 women screened, 789 (17.5%) were eligible and 551 women (70% of eligible) were enroled in the study. Of the 551 women enroled, data on delivery outcomes were missing for 37 (6.5%) women, and 36 (6.5%) experienced either an induced or spontaneous abortion. Out of the remaining 478 women, 468 had live births and 10 had stillbirths. Fifty‐five of the 478 women had no or only one follow‐up visit and were excluded from the analysis set. Thus, the cohort analysis set included 423 women who gave birth (live birth or stillbirth) and had at least three gestational weight measurements available (Figure 1). The baseline sociodemographic characteristics of the women included in the analysis cohort were comparable to those who were excluded.

### 2.2 Data Collection in Early, Mid‐ and Late Pregnancy

The study commenced in January 2021; the recruitment was completed in March 2023, and the last delivery visit was in November 2023. Trained research staff collected data at the following time points during pregnancy: enrolment in the study before 12 weeks (‘early pregnancy’/baseline visit, v_1_), at 23 ± 1 weeks (‘mid‐pregnancy’, v_2_) and at 33 ± 1 weeks (‘late pregnancy’, v_3_). In addition, research staff took weekly body weight measurements close to the expected date of delivery at 36–37 weeks (v_4_) and 39–40 weeks (v_5_). A post‐delivery visit was conducted to collect information on delivery details from the participants' ANC cards and to measure the anthropometrics of the neonates.

Sociodemographic (age, education, occupation/working status and religion of the participant) and lifestyle information and obstetric and gynaecological history were collected in a face‐to‐face interview during the baseline visit. The date of the last menstrual period (LMP) available in the ANC card was obtained and confirmed using the ultrasound reports available at the clinic. Length of gestation was calculated using the date of LMP.

The height of the women was measured to the nearest cm using a portable stadiometer (Seca model 213, Hamburg, Germany) during the baseline visit. Weight was measured to the nearest 100 g using calibrated weighing scales at all visits (v_1_–v_5_). The early‐pregnancy/baseline BMI (kg/m^2^) (a proxy for pre‐pregnancy) was calculated based on the first measured weight and height during early pregnancy and classified according to Asian‐specific BMI cut‐off points (WHO Expert Consultation 2004). Mid‐upper arm circumference (MUAC) was measured using a Seca tape to the nearest 1 mm (Seca 201). Total GWG was calculated by subtracting the weight measured at v_1_ from the final prenatal weight on the ANC card. The latest available weight measured by the research team, within 28 days prior to the delivery, was used to calculate total GWG in case of non‐availability of final prenatal weight on the ANC card. Details of birthdate and birthweight were obtained from the ANC card. Neonatal length was measured using an infantometer and neonatal head circumference was measured using a non‐stretchable tape (Seca 201) within 28 days of delivery.

### 2.3 Exposure and Outcome Variables

Maternal weights measured at different time points during pregnancy were used to explore weight trajectories. Rates of GWG (kg/week) were calculated as follows: (1) between v_1_ and v_2_: the ratio of the difference between the weight at v_2_ and v_1_ to the difference in gestational length at v_2_ and v_1_, (2) between v_2_ and v_3_: the ratio of the difference between the weight at v_3_ and v_2_ to the difference in gestational length at v_3_ and v_2_ and (3) between v_1_ and v_3_: as the ratio of the difference between the weight at v_3_ and v_1_ to the difference in gestational length at v_3_ and v_1_.

The gestational age at delivery was calculated from the date of the LMP to the delivery date. LBW was defined as newborns weighing < 2.5 kg (World Health Organisation n.d.b) and preterm birth was defined as a delivery before 37 weeks of gestation (World Health Organisation n.d.b). Macrosomia was defined as a birthweight > 4.0 kg (Harvey et al. 2021). The size of the newborn was compared to the international standard for the newborn weight from the INTERGROWTH‐21st Project (Villar et al. 2014). Based on these reference standards, newborns weighing less than 10th percentile and above 90th percentile for their gestational age were categorised as SGA and LGA, respectively.

### 2.4 Statistical Analyses

Data are presented as the sample mean with 95% confidence interval (95% CI) and median and interquartile range (*Q*1, *Q*3) values for continuous variables and number (% with 95% CI) for categorical variables. The median total GWG and the rate of GWG between v_1_ and v_2_, v_2_ and v_3_ and v_1_ and v_3_ were assessed in the baseline BMI categories. The distribution of neonatal outcomes in BMI categories is presented in Table S1.

Statistical analysis was approached in two steps: (1) longitudinal cluster analysis and (2) regression analysis of neonatal outcomes given the trajectory clusters.

Longitudinal clustering using maternal weights: To capture the information available in the maternal baseline weight and changes in the weight during the pregnancy, longitudinal cluster analysis was performed. Clustering longitudinal data approximates the heterogeneity in terms of clusters and the clusters can be used in further analyses (Den Teuling 2023). A longitudinal clustering technique finds groups based on between‐person differences accounting for within‐person changes over time (Den Teuling 2023).

As mentioned earlier, the weights of women were measured at irregular weeks within each interval and not all 423 women had weight measurements for all five visits. Therefore, we imputed weights at the following time points (12, 23, 33, 36 and 39 gestational weeks) using the following statistical procedure. We assumed that the missingness of weight measurements was completely random because it was not related to any data (observed or unobserved).

First, the irregularly measured weight data set was converted into a set of repeated measures at selected time points, that is, 12, 23, 33, 36 and 39 gestational weeks using a broken stick model, which is the linear mixed model, with time as a linear B‐spline and with a participant as grouping factor (Van Buuren 2023). The adequacy of model fitting was assessed by visually inspecting the fitted weights against observed weights at the subject level, and by checking the range (0, 1) of all elements of the design matrix (Van Buuren 2023). The fitted weights at the five‐time points were used for longitudinal clustering. The Linear regression K‐means (LMKM) approach was employed to obtain maternal weight trajectories. It is a two‐step approach where, in the first step, each trajectory was modelled using linear regression for a woman; in the second step, the K‐means algorithm was used to cluster the subject parameter estimates. Each woman was classified into one cluster. Cluster results were assessed by using convergence of the cluster solution, by plotting the weight trajectories and visually checking whether the identified patterns were sufficiently distinct and clinically meaningful. In addition, we plotted residuals and inspected them visually using *Q*–*Q* plots. Dunn's index (higher is better), Bayesian Information Criterion (lower is better) and estimation time (seconds) were used to decide a model with an optimal number of clusters. We restricted the number of clusters to 3, 4 or 5. We repeated the cluster analysis using Growth Mixture Modelling through latent‐class mixed modelling. The similarity between the cluster partition of the two methods was assessed using the Adjusted Rand Index (ARI) (Hubert and Arabie 1985). Results of the model diagnostic of LMKM and ARI are presented in Table S2.

Thus, the longitudinally collected gestational weights from v_1_ to v_5_ were used for building the weight trajectories and their clusters. Weights collected at v_1_, v_2_ and v_3_ were used to calculate the rate of GWG between v_1_ and v_2_, v_2_ and v_3_ and v_1_ and v_3_. We characterised the clusters using baseline BMI categories and rates of GWG.

Trajectory clusters and adverse neonatal outcomes: Multivariable logistic regression analyses were performed to study the association of trajectory clusters with the neonatal outcomes (LBW, SGA and preterm birth) and adjusted odds ratio (aOR) with 95% CI were reported. The following explanatory variables were used in the regression analyses: trajectory clusters (Cluster 1 as a reference category for analysis purposes), participant's age, height, parity, education, working status and religion. The model for LBW was additionally adjusted for the length of gestation at delivery.

Analyses were carried out using R 4.3.3 (packages: broken stick, LAtrend, LCMM and glm packages) and SAS v 9.4.

### 2.5 Ethical Statement

Written informed consent was obtained from an eligible woman after she was explained and had understood the participant information sheet. If an eligible woman was illiterate, a thumb impression was taken from her after ensuring that she had understood and had also explicitly stated her consent verbally; a literate impartial witness signed the consent form on her behalf. Ethics approvals were obtained from the Ethics Committee of Jehangir Clinical Development Centre Pvt Ltd. (EC Registration No: ECR/352/lnst/MH/2013/RR‐19) and the Ethics Committee of the Tampere Region. All study procedures were conducted according to the guidelines in the Declaration of Helsinki.

## 3 Results

### 3.1 Baseline Characteristics

The baseline characteristics of 423 study participants are presented in Table 2. The mean age was 25 ± 4.5 years, 60% were Hindus and 55% were nulliparous. One‐third of the women had completed at least 8 years of education, and 86% were homemakers. The median baseline weight, height and MUAC were 50 (*Q*1, *Q*3: 44, 58) kg, 153.4 (*Q*1, *Q*3: 149.6, 157.5) cm and 25.0 (*Q*1, *Q*3: 22.5, 27.8) cm, respectively. The prevalence of underweight (24.6%, 95% CI, 21, 29) and overweight (26.5%, 95% CI, 22, 31) were similar, and 11.0% (95% CI, 8, 14) of women were obese.

### 3.2 Rates of GWG and Total GWG by Baseline BMI Categories

Weight measurements at v_1_ were available for 423 women, at v_2_ for 405 (96%), at v_3_ for 412 (97%), at v_4_ for 368 (87%) and at v_5_ for 308 (73%) women. The median rate of weight gain increased with increasing length of gestation in all BMI categories. The median gestational age was 39.4 (*Q*1, *Q*3: 38.4, 40.1) weeks and the median total GWG was 10.3 (*Q*1, *Q*3: 7.8, 13.4) kg at delivery. The median rates (v_1_–v_2_, v_2_–v_3,_ v_1_–v_3_) of GWG, and total GWG were highest among the underweight and least among the obese women (Table 1).

**Table 1:** Baseline BMI categories and rate of GWG in mid‐, late, early‐late pregnancy and total GWG (*n* = 423).

	*n*	Rate of GWG in mid‐pregnancy (v_1_–v_2_) (kg/week)	Rate of GWG in late pregnancy (v_2_–v_3_) (kg/week)	Rate of GWG in early‐late pregnancy (v_1_–v_3_) (kg/week)	Total GWG (kg)
Underweight (< 18.5 kg/m^2^)	104	0.34 (0.21, 0.42)	0.41 (0.29, 0.55)	0.37 (0.31, 0.46)	11.7 (8.6, 13.7)
Normal weight (< 23.0 kg/m^2^)	161	0.26 (0.15, 0.40)	0.43 (0.27, 0.57)	0.33 (0.24, 0.44)	11.0 (8.5, 14.3)
Overweight (< 27.5 kg/m^2^)	112	0.18 (0.04, 0.33)	0.30 (0.18, 0.26)	0.26 (0.14, 0.35)	8.9 (5.9, 11.5)
Obesity (≥ 27.5 kg/m^2^)	46	0.08 (−0.08, 0.23)	0.29 (0.12, 0.39)	0.19 (0.04, 0.31)	7.8 (3.7, 11.3)
Total	423	0.24 (0.11, 0.38)	0.39 (0.24, 0.52)	0.32 (0.21, 0.42)	10.3 (7.8, 13.4)

*Note:* Values are median (*Q*1, *Q*3). *p* values indicate a linear trend of rates of GWG and total GWG across the increasing BMI categories, that is, underweight, normal weight, overweight and women with obesity.

Abbreviations: GWG, Gestational weight gain; v1, < 12 weeks; v2, 23 ± 1 weeks, v3, 33 ± 1 weeks.

### 3.3 Characterisation of Weight Trajectory Clusters

**Figure 2:**
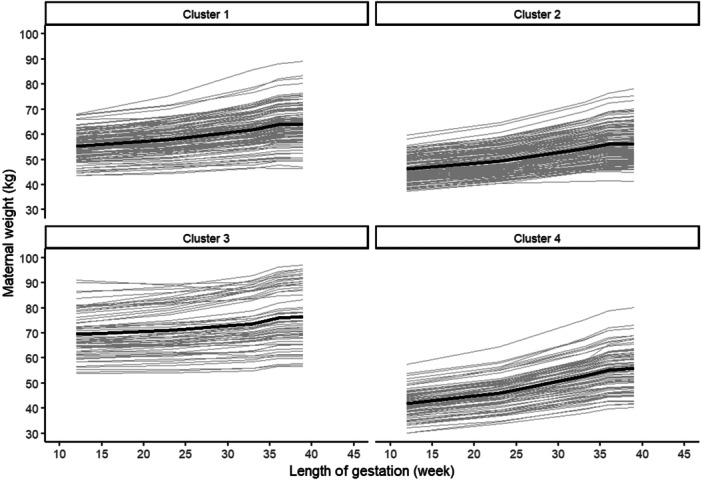
Trajectory clusters using maternal weight measurements during pregnancy.

Figure 2 and Table 2 represent the four distinct trajectory clusters of maternal weight. The mean baseline weight of Cluster 1 (29% of the cohort) was 53.6 (95% CI, 52.7, 54.5) kg; the mean rate of GWG was 0.27 (95% CI, 0.24, 0.30) kg/week from v1 to v3 and the mean total GWG 9.2 (95% CI, 8.6, 9.8) kg. In Cluster 1, around 97% of women were either normal or overweight and the mean BMI was 22.9 (95% CI, 22.5, 23.3) kg/m^2^. Cluster 2 comprised 35% of the total cohort, with a mean baseline weight of 46.3 (95% CI, 45.6, 47.1) kg and a mean rate of GWG of 0.31 (95% CI, 0.28, 0.34) kg/week from v1 to v3. These women had a mean baseline BMI of 19.4 (95% CI, 19.0, 19.8) kg/m^2^, and around 93% of them were either underweight or normal weight. Cluster 3 comprised 16% of the women who had an average GWG rate of 0.17 (95% CI, 0.12, 0.22) kg/week in v1–v3. The women in this group were the tallest 155.5 (95% CI, 154.1, 156.9) and with a higher mean BMI of 29.1 (95% CI, 28.1, 30.1) kg/m^2^ compared to other clusters. All women were either overweight or obese at baseline and they had the lowest mean total GWG 6.4 (95% CI, 5.2, 7.6) kg among all clusters. Cluster 4 comprised 20% of the total cohort. The mean BMI of the women in this group was 18.0 (95% CI, 17.6, 18.5) kg/m^2^ and all women were either underweight or normal weight at baseline. The average GWG rate was 0.47 (95% CI, 0.44, 0.50) kg/week from v1 to v3 and had the highest total GWG 15.5 (95% CI, 14.7, 16.3) kg in the cohort. The distribution of the baseline BMI and total GWG in each cluster are shown in Figure S1.

**Table 2:** Sociodemographic and anthropometric characteristics of women by trajectory clusters and in full cohort (*n* = 423).

		Trajectory clusters				
		Cluster 1	Cluster 2	Cluster 3	Cluster 4	Total
*n* (%)		124 (29%)	146 (35%)	68 (16%)	85 (20%)	423
Age		25.6 (24.8, 26.4)	24.2 (23.5, 24.9)	26.8 (25.7, 28.1)	22.6 (21.9, 23.3)	24.7 (24.3, 25.1)
Education, *n* (%)	Middle school certification or below	43 (34.7)	39 (26.7)	27 (39.7)	24 (28.2)	133 (31.4)
		(26.3, 43.1)	(19.5, 34.7)	(28.1, 51.3)	(18.7, 37.8)	(27.0, 36.1)
	High school to higher secondary certificate	63 (50.8)	83 (56.9)	34 (50.0)	50 (58.8)	230 (54.4)
		(42.0, 59.6)	(48.8, 64.9)	(38.1, 61.9)	(48.4, 69.3)	(49.6, 59.1)
	Graduate degree and above or professional diploma	18 (14.5)	24 (16.4)	7 (10.3)	11 (12.9)	60 (14.2)
		(8.3, 20.7)	(10.4, 22.5)	(3.1, 17.5)	(5.8, 20.1)	(10.9, 17.5)
Religion, *n* (%)	Hindu	67 (54.0)	100 (68.5)	30 (44.1)	56 (65.9)	253 (59.8)
		(45.3, 62.8)	(61.0, 76.0)	(32.3, 55.9)	(55.8, 75.9)	(55.1, 64.5)
	Muslim	51 (41.1)	34 (23.3)	32 (47.1)	26 (30.6)	143 (33.8)
		(32.5, 49.8)	(16.4, 30.1)	(35.2, 58.9)	(20.8, 40.4)	(29.3, 38.3)
	Others	6 (4.8)	12 (8.2)	6 (8.8)	3 (3.5)	27 (6.4)
		(1.1, 8.6)	(3.8, 12.7)	(2.1, 15.6)	(0, 7.5)	(4.1, 8.7)
Occupation, *n* (%)	Homemakers	105 (84.7)	124 (84.9)	57 (83.8) (75.1, 92.6)	79 (92.9)	365 (86.3)
		(78.3, 91.0)	(79.1, 90.7)		(87.5, 98.4)	(83.0, 89.6)
	Working	19 (15.3)	22 (15.1)	11 (16.2)	6 (7.1)	58 (13.7)
		(8.9, 21.7)	(9.3, 20.9)	(7.4, 24.9)	(1.6, 12.5)	(10.4, 16.9)
Monthly family income (INR)^a^		20,000 (15,000, 35,000)	20,000 (13,000, 38,000)	20,000 (15,000, 30,750)	18,000 (11,750, 25,000)	20,000 (14,000, 30,000)
Parity, *n* (%)	Nulliparous	60 (48.4)	80 (54.8)	29 (42.6)	62 (72.9)	231 (54.6)
		(39.6, 57.2)	(46.7, 62.9)	(30.9, 54.4)	(63.5, 82.4)	(49.9, 59.4)
	Primiparous	50 (40.3)	59 (40.4)	24 (35.3)	19 (22.4)	152 (35.9)
		(31.7, 48.9)	(32.5, 48.4)	(23.9, 46.7)	(13.5, 31.2)	(31.4, 40.1)
	Multiparous	14 (11.3)	7 (4.8)	15 (22.0)	4 (4.7)	40 (9.5)
		(5.7, 16.9)	(1.3, 8.3)	(12.2, 31.9)	(0.2, 9.2)	(6.7, 12.2)
Baseline weight (kg)^a^		53.6 (52.7, 54.5)	46.3 (45.6, 47.1)	70.4 (67.9, 72.9)	42.5 (41.3, 43.7)	52.0 (50.9, 53.1)
		53.0 (50.4, 57.0)	45.4 (43.0, 50.1)	68.0 (64.4, 76.5)	41.5 (38.0, 45.3)	50.3 (44.0, 57.6)
Height (cm)		153.6 (152.7, 154.5)	152.9 (152.0, 153.8)	155.5 (154.1, 156.9)	152.7 (151.5, 153.9)	153.5 (152.9, 154.1)
Baseline BMI (kg/m^2^)^a^		22.9 (22.5, 23.3)	19.4 (19.0, 19.8)	29.1 (28.1, 30.1)	18.0 (17.6, 18.5)	22.3 (21.9, 22.7)
		23.7 (21.8, 25.1)	19.9 (17.9, 21.2)	28.0 (25.9, 30.7)	17.9 (16.5, 19.2)	21.6 (18.6, 24.7)
Baseline BMI categories, *n* (%)	Underweight	0	59 (40)	0	53 (62)	104 (24.6)
	< 18.5 kg/m^2^		(33, 48)		(52, 73)	(21, 29)
	Normal weight	50 (40)	77 (53)	0	32 (38)	161 (38.1)
	18.5–23.0 kg/m^2^	(32, 49)	(45, 61)		(27, 48)	(33.4, 42.7)
	Overweight	70 (57)	10 (7)	26 (38)	0	112 (26.5)
	23.0–27.5 kg/m^2^	(48, 65)	(2, 8)	(27, 49)		(22, 31)
	Obese	4 (3)	0	42 (62)	0	46 (10.9)
	> 27.5 kg/m^2^	(1, 6)		(50, 73)		(8, 14)
Baseline mid‐upper arm circumference (cm)		25.9 (25.5, 26.3)	23.8 (23.4, 24.2)	31.0 (30.1, 31.9)	22.1 (21.6, 22.6)	25.3 (24.9, 25.7)
Rate of GWG (kg/week)^a^	Mid‐pregnancy (v_1_–v_2_)	0.21 (0.17, 0.25)	0.26 (0.21, 0.31)	0.10 (0.10, 0.15)	0.45 (0.41, 0.49)	0.25 (0.23, 0.27)
		0.20 (0.07, 0.33)	0.26 (0.15, 0.37)	0.08 (−0.04, 0.23)	0.38 (0.27, 0.49)	0.24 (0.1–0.4)
Rate of GWG (kg/week)^a^	Late pregnancy (v_2_–v_3_)	0.32 (0.28, 0.36)	0.38 (0.33, 0.43)	0.22 (0.15, 0.29)	0.59 (0.55, 0.63)	0.40 (0.38, 0.42)
		0.30 (0.21, 0.43)	0.41 (0.29, 0.53)	0.21 (0.09, 0.36)	0.55 (0.43, 0.67)	0.39 (0.2, 0.5)
Rate of GWG (kg/week)^a^	Early‐late pregnancy (v_1_–v_3_)	0.27 (0.24, 0.30)	0.31 (0.28, 0.34)	0.17 (0.12, 0.22)	0.47 (0.44, 0.50)	0.31 (0.29, 0.33)
		0.27 (0.18, 0.34)	0.31 (0.26, 0.39)	0.14 (0.03, 0.30)	0.47 (0.38, 0.56)	0.32 (0.21, 0.42)
Total GWG (kg)^a^	Early pregnancy to delivery	9.2 (8.6, 9.8)	10.6 (10.1, 11.1)	6.4 (5.2, 7.6)	15.5 (14.7, 16.3)	10.5 (10.1, 11.0)
		9.1 (7.0, 11.0)	10.6 (8.0, 12.7)	5.7 (2.7, 9.5)	14.9 (12.2, 17.8)	10.3 (7.6, 13.4)
Length of gestation at delivery (week)^a^		39.4 (38.6, 40.3)	39.1 (38.2, 40.1)	39.6 (38.5, 40.1)	39.7 (38.8, 40.3)	39.4 (38.4, 40.1)

*Note:* Values are mean (95% CI) or *n* (%, 95% CI).

Abbreviations: BMI, Body mass index; GWG, Gestational weight gain; MUAC, Mid‐upper arm circumference.

^a^ Median (*Q*1, *Q*3) are additionally provided.

### 3.4 Neonatal Outcomes by Weight Trajectory Clusters and the Baseline BMI Categories

Table 3 presents neonatal outcomes in the cohort and in the four clusters. Out of 423 total births, 416 (98%) were live births and the rest were stillbirths. The birthweight and gender of the stillbirths were not available. Among the live births, the mean birthweight was 2.8 (95% CI, 2.76, 2.84) kg, the mean neonatal length was 49.0 (95% CI, 48.8, 49.2) cm and the mean neonatal head circumference was 33.1 (95% CI, 32.9, 33.3) cm. Seventy‐four newborns (18%, 95% CI, 14, 22) were LBW and 0.5% (95% CI, 0, 1, *n* = 2) had macrosomia. Around 44% (95% CI, 40, 49, *n* = 185) of newborns were SGA and 2% (95% CI, 1, 3, *n* = 8) were LGA. Around 10% (95% CI, 7, 13, *n* = 40) were preterm births. Nine newborns were both SGA and preterm, and 6 (4%) of these were in Cluster 2. The mean birthweight was similar in Clusters 3 and 4 (2.9, 95% CI, 2.8, 3.0 kg and 2.8, 95% CI, 2.7, 2.9 kg), and the lowest in Cluster 2 (2.7, 95% CI, 2.6, 2.8 kg). The mean neonatal length and head circumference were similar in all clusters. The women in Cluster 2 had 54% (95% CI, 46, 63) of SGA and 23% (95% CI, 16, 30) of LBW newborns. Fifteen percent (95% CI, 6, 23) of babies born to women in Cluster 3 were preterm, followed by 13% (95% CI, 8, 19) in Cluster 2. The percentage of women with caesarean section (C‐section) was similar in all the clusters.

**Table 3:** Neonatal outcomes by trajectory clusters and in full cohort (*n* = 423).

	Trajectory clusters				
	Cluster 1	Cluster 2	Cluster 3	Cluster 4	Total
*n* (%)	124 (29%)	146 (35%)	68 (16%)	85 (20%)	423
Stillbirths	1 (1)	3 (2)	1 (1)	2 (2)	7 (2)
Live births	123 (99)	143 (98)	67 (99)	83 (98)	416 (98)
Boys (%)	63 (51)	75 (51)	32 (48)	47 (54)	217 (52)
	(42, 59)	(43, 59)	(37, 59)	(44, 64)	(47, 57)
Birth weight (kg)	2.9 (2.8, 3.0)	2.7 (2.6, 2.8)	2.9 (2.8, 3.0)	2.8 (2.7, 2.9)	2.8 (2.76, 2.84)
Neonatal length (cm)	49.0 (48.5, 49.5)	48.9 (48.5, 49.3)	48.9 (48.3, 49.5)	49.4 (48.9, 49.9)	49 (48.8, 49.2)
Head circumference (cm)	33.2 (32.9, 33.5)	33.1 (32.9, 33.3)	33.2 (32.8, 33.6)	33.1 (32.7, 33.5)	33.1 (32.9, 33.3)
LBW newborns, *n* (%)	17 (14)	33 (23)	9 (13)	15 (18)	74 (18)
	(8, 21)	(15, 31)	(6, 20)	(12, 24)	(14, 22)
Birth weight > 3.5 kg, *n* (%)	11 (9)	6 (4)	6 (9)	2 (2)	25 (6)
	(4, 14)	(1, 7)	(2, 16)	(0, 6)	(4, 8)
Newborns with macrosomia, *n* (%)	1 (1)	1(1)	0	0	2 (0.5)
	(0, 4)	(0, 2)			(0, 1)
SGA newborns, *n* (%)	42 (34)	77 (54)	27 (40)	39 (46)	185 (44)
	(26, 42)	(46, 63)	(35, 45)	(41, 51)	(40, 49)
LGA newborns, *n* (%)	0 (0)	3 (2)	4 (6)	1 (1)	8 (2)
		(0, 4)	(1, 11)	(0, 4)	(1, 3)
Preterm birth, *n* (%)	6 (5)	19 (13)	10 (15)	5 (6)	40 (10)
	(1, 8)	(8, 19)	(6, 23)	(1, 11)	(7, 13)
Mode of delivery (caesarean section)	49 (40)	59 (40)	26 (39)	24 (29)	158 (38)
	(31, 48)	(32, 49)	(27, 51)	(19, 40)	(33, 43)
SGA newborns and preterm birth, *n* (%)	1 (1)	6 (4)	1(2)	1 (1)	9 (2)
	(0, 2)	(1, 7)	(0, 4)	(0, 4)	(1, 4)
SGA and LBW newborns, *n* (%)	15 (12)	30 (21)	7 (10)	15 (18)	67 (16)
	(6, 18)	(14, 27)	(3, 18)	(10, 26)	(13, 20)

*Note:* Values are mean (95% CI) or *n* (%, 95% CI); gender and birthweight were available among live births.

Abbreviations: LBW, Low birth weight (birthweight < 2.5 kg); LGA, Large for gestational age (> 90th percentile as per INTERGROWTH‐21st standards); Macrosomia, birthweight > 4.0 kg; Preterm birth, < 37 weeks of gestation; SGA, Small for gestational age (< 10th percentile as per INTERGROWTH‐21st standards).

When neonatal outcomes were assessed as per women's baseline BMI categories, 23% (95% CI, 14, 30) babies born to underweight women were LBW, 56% (95% CI, 44, 63) were SGA and 5% (95% CI, 1, 9) were preterm births whereas 11% (95% CI, 2, 20) were LBW, 39% (95% CI, 25, 53) were SGA and 13% (95% CI, 4, 22) were preterm births among women with obesity. Twenty‐eight percent (95% CI, 19, 37) of underweight and 47% (95% CI, 31, 46) of obese women underwent C‐section deliveries (Table S1).

### 3.5 Weight Trajectory Clusters and Risk of Adverse Neonatal Outcomes

The mean baseline weight, height and BMI of women in Cluster 1 and that of the full cohort were similar. Therefore, Cluster 1 was used as a reference cluster in regression analyses. None of the clusters were associated with the likelihood of having an LBW newborn but being tall (height > 150 cm) was inversely associated with it. Women in Cluster 2 were at a higher risk of having SGA and having preterm newborns compared to the women in Cluster 1. Women in Cluster 4 were not at higher risk of having SGA and/or preterm newborns despite their lower baseline BMI status than that of women in Cluster 2. Women in Cluster 3 had a higher risk of preterm births. All the models were adjusted for maternal age, education, religion, working status and parity. The interaction between clusters and working status was tested because the risk of having preterm births was higher among working women compared to homemakers. The interaction term was nonsignificant and the relationship between Cluster 2 (aOR: 3.8, 95% CI, 1.2, 17.1) and Cluster 3 (aOR: 5.1, 95% CI, 1.3, 20.8), with the risk of having preterm newborns compared to Cluster 1 remained unchanged (results not shown) (Table 4).

**Table 4:** Adjusted odds ratio and 95% CI for associations between trajectory clusters and adverse neonatal outcomes.

	Low birth weight^a^		Small for gestational age		Preterm birth	
Covariates	AOR	(95% CI)	AOR	(95% CI)	AOR	(95% CI)
Trajectory clusters						
Cluster 1	1		1		1	
Cluster 2	1.42	(0.71, 2.92)	**2.18**	**(1.31, 3.67)**	**2.95**	**(1.14, 8.73)**
Cluster 3	0.99	(0.38, 2.51)	1.35	(0.71, 2.53)	**3.99**	**(1.37, 12.71)**
Cluster 4	1.60	(0.70, 3.67)	1.75	(0.90, 3.22)	1.23	(0.32, 4.52)
Maternal height						
< 150 cm	1		1		1	
≥ 150 cm	**0.40**	**(0.23, 0.70)**	**0.58**	**(0.37, 0.90)**	0.60	(0.29, 1.26)

*Note:* All models include the following covariates (clusters, height, age, parity, education, working status, religion of the participants).

Abbreviations: AOR, Adjusted odds ratio; CI, Confidence interval; LBW, Birthweight < 2.5 kg; Preterm birth, < 37 weeks of gestation; SGA, < 10th percentile as per INTERGROWTH‐21st standards.

^a^ The model additionally adjusted for the length of gestation.

## 4 Discussion

### 4.1 The Main Results

The mean rates of GWG at mid‐ and late pregnancy, as well as the total GWG, were highest among underweight and lowest among obese women. GWG rates increased with gestational age across all BMI categories. The four distinct trajectory clusters summarised the variability in maternal weights, and we expressed them in terms of the baseline BMI and the rate of GWG. The clusters were characterised by women with (i) similar early‐pregnancy weights but different rates of GWG, (ii) different early‐pregnancy weights but comparable rates of GWG and (iii) apparently higher pre‐pregnancy weights but minimal weight gain during pregnancy. The clusters provided a coherent way to model longitudinal data in further regression analyses. Women in Cluster 2 (underweight and normal weight women), with a mean rate of GWG of 0.31 kg/week, were at a higher risk of delivering SGA preterm babies compared to the women in Cluster 1. Women in Cluster 3 (overweight and obese women), with a mean rate of GWG of 0.17 kg/week, were at a higher risk of preterm births compared to women in Cluster 1.

### 4.2 Comparison of the Results With the Previous Literature

The mean total GWG in our study aligned with the mean GWG reported in other Indian studies (Patel et al. 2023). The mean GWG in overweight/obese women was significantly lower compared to underweight or normal‐weight women, which is in line with other regional and international studies (Aji et al. 2022; Dangat et al. 2022). Very few Indian studies have reported rates of GWG in different periods of pregnancy. Bauserman et al. (2021) observed the rate of GWG of 0.39 ± 0.13 kg/week between 12 and 32 weeks (mean baseline BMI 20.0 ± 3.4 kg/m^2^) among 590 women from rural India, which was higher than what was observed in our study.

In our study, 18% (95% CI, 14, 22) of babies were LBW, which was higher than the global average of 15% (Unicef 2024) and the result in the slum study in South India (15%) (Mamidi et al. 2022; Unicef 2024) but similar to the results reported by a national study (18%) (Jana 2023). The overall incidence of SGA was 44% (95% CI, 40, 49) in our study, aligning with a reported range of 8% to 48% in studies from different regions of India (Mamidi et al. 2022; Rai et al. 2019; Sebastian et al. 2015). In Cluster 2, 54% (95% CI, 46, 63) of women had an SGA baby and 23% (95% CI, 15, 31) of women had an LBW baby. These women had a mean BMI of 19 kg/m^2^; they belonged to either the underweight or normal weight category with an average height of 153 cm. The rate of GWG was 0.31 kg/week from v1–v3 and the total mean GWG was 10.6 (95% CI, 10.1, 11.1) kg among these women. It is likely that smaller maternal size combined with a lower rate of GWG has contributed to the greater percentage of SGA newborns and preterm births in this group (Han et al. 2011; Kozuki et al. 2015). These women could have been targeted for intervention based on their baseline BMI and regular monitoring of GWG rates. It is worth noting that 60% (37 of 77) of SGA newborns had normal birthweight. In low‐resource settings, newborns often have a normal birthweight but are SGA due to factors such as low maternal height and weight and ethnicity, and do not have an increased risk of perinatal mortality and morbidity (Malin et al. 2014).

In our study, 10% (95% CI, 7, 13) were preterm births, consistent with the results (12% preterm births) of a national survey‐based study (Jana 2023). The women in Cluster 3 had a higher risk of preterm births compared to the women in Cluster 1. Cluster 3 included overweight and obese, comparatively older women in the cohort, having the lowest rate of weight gain. Nearly 60% of the women in this group were parous. Maternal overweight and obesity, increasing parity and age are known risk exposures for preterm birth (Cornish et al. 2024; Vats et al. 2021). In Cluster 4, women were young, nearly 75% were nulliparous, 60% were underweight and 40% were normal weight at baseline, and they had the highest rate of GWG in mid‐ and late pregnancy compared to women in the other clusters. The percentage of SGA and/or preterm newborns and the percentage of women undergoing C‐sections were relatively lower in this cluster despite having lower weight, similar MUAC and height profile at baseline as in Cluster 2. This implies the importance of appropriate GWG in relation to the baseline BMI. A China‐based cohort study has reported the protective role of high GWG (0.5 kg/week) among underweight women against the risk of SGA babies (Wei et al. 2022). The women in Clusters 2 and 4 differed in the rate of GWG and total GWG. The disparity between rates of GWG and total GWG might be attributable to their parity as Cluster 2 had a higher percentage of parous women compared to Cluster 4. Higher parity might be a driving factor of lower GWG as seen in other national and international studies (Dangat et al. 2022; Darling et al. 2023; Thiruvengadam et al. 2022). Women in Clusters 1 and 2 differed in initial weights, BMI categories and MUACs. However, they gained weight almost at similar rates (0.27 [0.18, 0.34] vs. 0.31 [0.26, 0.39] kg/week). This could be one of the reasons for having a higher percentage of SGA babies in Cluster 2 compared to Cluster 1. Thus, appropriate GWG as per baseline BMI status is important. In addition to differences in early pregnancy BMI, the differences in GWG rates and total GWG between different clusters may be attributable to several reasons such as dietary intake, physical activity or maternal morbidity. Therefore, these results should be interpreted cautiously and warrant further analyses.

### 4.3 Strengths and Weaknesses

This study has several strengths. Our study assessed the combined effect of baseline BMI and GWG by employing a data‐driven analytical approach using a sound statistical technique that leveraged the efficient use of longitudinal gestational weight data. The broken stick model used a mixed model approach for longitudinal weight measurements collected at different weeks within the visit intervals to minimise bias from timepoint binning (Van Buuren 2023). The longitudinal clustering technique identified four distinct clinically meaningful weight trajectory clusters. The results with the LMKM method were presented because it is suitable when only a few observations per participant are available (Den Teuling 2023). The applicability of IOM guidelines for GWG in India is limited due to differences in body size and cut‐offs for BMI classification (WHO Expert Consultation 2004). This analytical approach works well where standard guidelines of GWG are not available. The methods developed here are applicable to other similar studies with longitudinal gestational weight data. Given the pre‐pregnancy and first‐trimester weight, we can predict the weight trajectory for the later part of the pregnancy and predict the risk of adverse neonatal outcomes. However, to ensure the generalisability of this approach, larger‐scale studies are necessary. The regression analyses were adjusted for important confounders. However, the possibility of residual confounding cannot be excluded. This study also attempts to fill the gap in the maternal health literature related to trimester‐specific rates of GWG among slum‐dwelling women in India. This prospective cohort study provided direct measurements of key variables, including serial maternal weights taken by trained staff during each antenatal visit, thereby reducing recall and misclassification bias.

Our study was not without limitations. Even though the generalisability of the results may be limited as the study was conducted in a single city in India, the mean GWG and rate of GWG observed were consistent with previous studies (Bauserman et al. 2021; Dangat et al. 2022; Patel et al. 2023). Ideally, baseline weight and BMI classification would be based on pre‐pregnancy weight, but relying on self‐reported pre‐pregnancy weight in our study was not feasible. Pregnancy outcomes were unavailable for 37 women, and 55 women had no or only one follow‐up visit, resulting in the analysis set of 423 women. These findings reiterate the importance of early registration of pregnancy and increasing its awareness among slum‐dwelling women.

## 5 Conclusion

Four clinically relevant clusters summarised longitudinal trajectories of maternal weights during pregnancy. The clusters were characterised by women with (i) similar early‐pregnancy weights but different rates of GWG, (ii) different early‐pregnancy weights but comparable rates of GWG and (iii) apparently higher pre‐pregnancy weights but minimal weight gain during pregnancy. These clusters were associated with the neonatal outcomes studied in this paper. Our findings emphasise the need to address both the pre‐pregnancy BMI and regular weight monitoring starting from early pregnancy, thereby providing a possibility for timely intervention to manage GWG for achieving favourable neonatal outcomes.

Further careful cluster‐based analysis, based on data from larger Indian studies, may enable the drafting of appropriate BMI‐specific recommendations on GWG for Indian women. Given pre‐pregnancy weight and first‐trimester weight information, it is possible to predict the weight trajectory for the later part of the pregnancy and predict the risk of adverse neonatal outcomes. Such predictive modelling can be valuable for counselling expectant mothers. However, to ensure the generalisability of this approach, larger‐scale studies are necessary. Studying the key determinants of GWG such as dietary intake and physical activity in large multicentric studies, and exploring different health, medical and cultural reasons behind the determinants would facilitate designing appropriate interventions.

## Author Contributions

Study conceptualisation: Swapna Deshpande and Sangita Kulathinal. Data collection and supervision: Rubina Mandlik, Swapna Deshpande and Anuradha Khadilkar. Statistical analysis and initial drafting of manuscript: Swapna Deshpande. Results interpretation, manuscript review, revisions and finalisation: Swapna Deshpande, Tarja I. Kinnunen, Rubina Mandlik, Anuradha Khadilkar, Suhas Otiv and Sangita Kulathinal. Final approval of the manuscript: Swapna Deshpande, Tarja I. Kinnunen, Rubina Mandlik, Anuradha Khadilkar, Suhas Otiv and Sangita Kulathinal.

## Conflicts of Interest

The authors declare no conflicts of interest.

## Supporting information

SUPPLEMENTARY TABLE 1 Neonatal outcomes by baseline BMI categories and in full cohort, (n=423). Footnote: Values are n (%), birthweights were available among livebirths. LBW: low birth weight (birthweight <2.5kg), macrosomia: birthweight >4.0 kg, SGA: small for gestational age (<10^th^ percentile as per INTERGROWTH‐21^st^ standards), LGA: Large for gestational age (>90^th^ percentile as per INTERGROWTH‐21^st^ standards), Preterm birth: <37 weeks of gestation.

SUPPLEMENTARY TABLE 2 Model diagnostics for selecting the optimum number of clusters using the LMKM method. Footnote: LMKM: Linear regression K‐means method. The adjusted Rand Index between the partitions done by LMKM and GMM methods is 0.647.

SUPPLEMENTARY FIGURE 1 Maternal baseline BMI and total gestational weight gain in each cluster.

## Data Availability

The data sets cannot be made publicly available because public availability would compromise participant privacy. The data sets used and analysed during the current study are available from the corresponding author upon reasonable request.
