# Supplementary material for: Association of Gestational Weight Trajectories With Neonatal Outcomes Among Pregnant Slum‐Dwelling Women, India
Source: Matern Child Nutr. 2025 Apr 28;21(3):e13805. doi: 10.1111/mcn.13805 (PMC12150156; doi:10.1111/mcn.13805)
Supplement: Supplementary file 1 — SUPPLEMENTARY TABLE 1 Neonatal outcomes by baseline BMI categories and in full cohort, (n=423). Footnote: Values are n (%), birthweights were available among livebirths. LBW: low birth weight (birthweight <2.5kg), macrosomia: birthweight >4.0 kg, SGA: small for gestational age (<10th percentile as per INTERGROWTH‐21st standards), LGA: Large for gestational age (>90th percentile as per INTERGROWTH‐21st standards), Preterm birth: <37 weeks of gestation. [file MCN-21-e13805-s002.docx]

SUPPLEMENTARY TABLE 1. Neonatal outcomes by baseline BMI categories and in full cohort, (n=423)

|  | **Baseline BMI categories** | | | | |
| --- | --- | --- | --- | --- | --- |
|  | **Underweight** | **Normal weight** | **Overweight** | **Obese** | **Total** |
| n (%) | 104 (25) | 161(38) | 112 (26) | 46 (11) | 423 |
| Stillbirths, n (%) | 2 (2) | 3 (2) | 1 (1) | 1 (2) | 7 (2) |
| Livebirths, n(%) | 102 (98) | 158 (98) | 111 (99) | 45 (98) | 416 (98) |
| LBW newborns, n (%) | 23 (23) | 34 (22) | 12 (11) | 5 (11) | 74 (18) |
| Newborns with macrosomia, n (%) | 0 | 2 (1) | 0 | 0 | 2 (0.5) |
| SGA newborns, n (%) | 56 (56) | 72 (46) | 39 (35) | 18 (40) | 185 (44) |
| LGA newborns, n (%) | 1 (1) | 3 (2) | 2 (2) | 2 (4) | 8 (2) |
| Preterm birth, n (%) | 5 (5) | 19 (12) | 10 (9) | 6 (13) | 40 (10) |
| Mode of delivery (Cesarean section) | 29 (28) | 63 (39.8) | 45 (40.5) | 21(46.7) | 158 (37) |
| SGA newborns and preterm birth, n (%) | 1(1) | 5 (3) | 2 (2) | 1(2) | 9 (2) |
| SGA and LBW newborns, n (%) | 21 (20) | 31(19) | 11 (10) | 4 (9) | 67 (16) |

Values are n (%), birthweights were available among livebirths. LBW: low birth weight (birthweight <2.5kg), macrosomia: birthweight >4.0 kg, SGA: small for gestational age (<10^th^ percentile as per INTERGROWTH-21^st^ standards), LGA: Large for gestational age (>90^th^ percentile as per INTERGROWTH-21^st^ standards), Preterm birth: <37 weeks of gestation.
