# Supplementary material for: Association of Gestational Weight Trajectories With Neonatal Outcomes Among Pregnant Slum‐Dwelling Women, India
Source: Matern Child Nutr. 2025 Apr 28;21(3):e13805. doi: 10.1111/mcn.13805 (PMC12150156; doi:10.1111/mcn.13805)
Supplement: Supplementary file 2 — SUPPLEMENTARY TABLE 2 Model diagnostics for selecting the optimum number of clusters using the LMKM method. Footnote: LMKM: Linear regression K‐means method. The adjusted Rand Index between the partitions done by LMKM and GMM methods is 0.647. [file MCN-21-e13805-s001.docx]

SUPPLEMENTARY TABLE 2. Model diagnostics for selecting the optimum number of clusters using the LMKM method

| LMKM method | Number of clusters | | | |
| --- | --- | --- | --- | --- |
|  | 2 clusters | 3 clusters | 4 clusters | 5 clusters |
| Dunn’s index | 6.77*10^-3^ | 0.0074 | 0.0123 | 0.098 |
| Bayesian Information Criteria (BIC) | 1767.8 | 1444.1 | 1195.4 | 1178.4 |
| Estimation time (sec) | 0.00000 | 0.0000 | 0.0000 | 0.0000 |

LMKM: Linear regression K-means method.

The adjusted Rand Index between the partitions done by LMKM and GMM methods is 0.647.
