# Supplementary figures and images for: Association of Gestational Weight Trajectories With Neonatal Outcomes Among Pregnant Slum‐Dwelling Women, India
Source: Matern Child Nutr. 2025 Apr 28;21(3):e13805. doi: 10.1111/mcn.13805 (PMC12150156; doi:10.1111/mcn.13805)

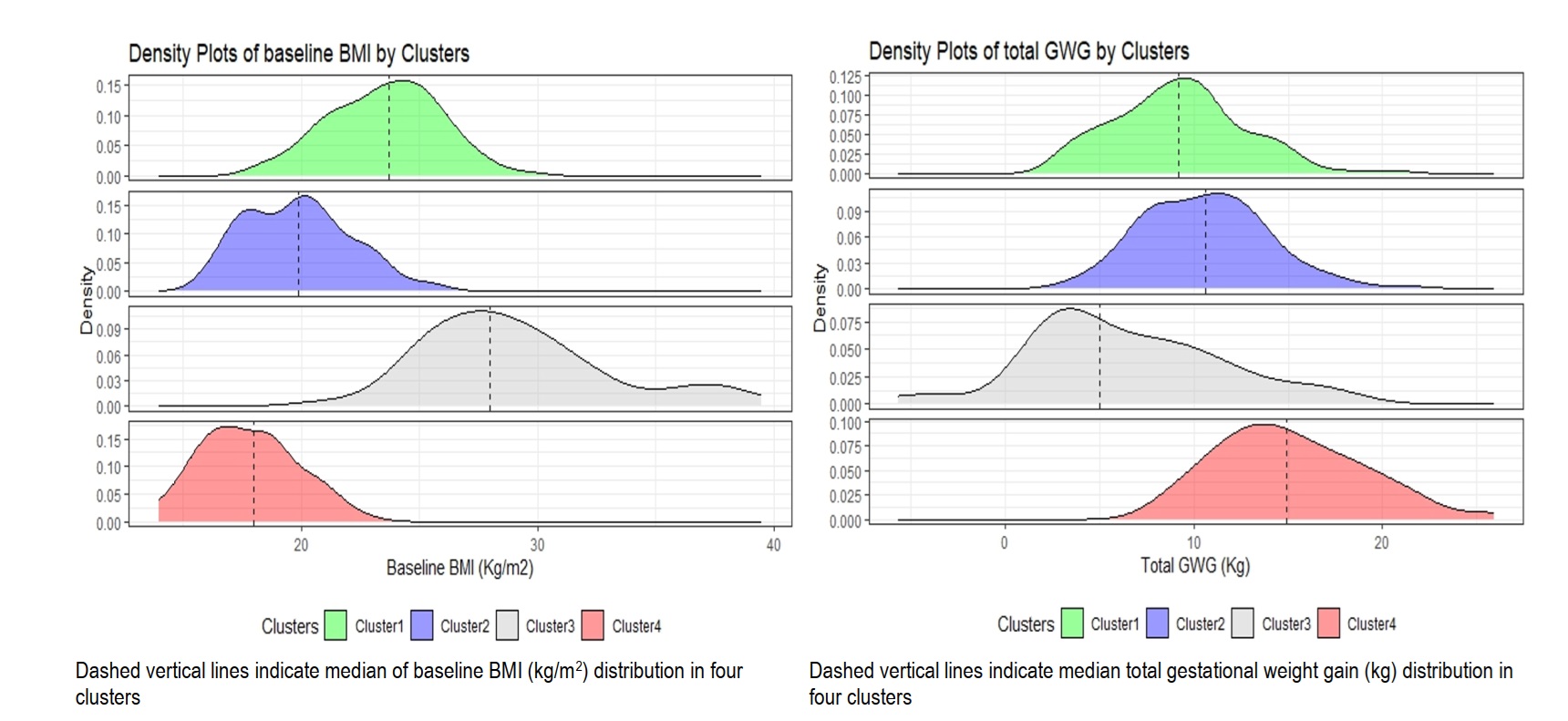

Supplement: Supplementary file 3 — SUPPLEMENTARY FIGURE 1 Maternal baseline BMI and total gestational weight gain in each cluster. [file MCN-21-e13805-s003.jpg]
